# Increase of Fungal Pathogenicity and Role of Plant Glutamine in Nitrogen-Induced Susceptibility (NIS) To Rice Blast

**DOI:** 10.3389/fpls.2017.00265

**Published:** 2017-02-28

**Authors:** Huichuan Huang, Thuy Nguyen Thi Thu, Xiahong He, Antoine Gravot, Stéphane Bernillon, Elsa Ballini, Jean-Benoit Morel

**Affiliations:** ^1^State Key Laboratory for Conservation and Utilization of Bio-Resources in Yunnan, Key Laboratory of Agro-Biodiversity and Pest Management of Education Ministry of China, Yunnan Agricultural UniversityKunming, China; ^2^Faculty of Agronomy, Hue University of Agriculture and ForestryHue, Vietnam; ^3^UMR1349 IGEPP, Université Rennes 1Rennes, France; ^4^INRA, UMR1332, Biologie du Fruit et Pathologie, Plateforme Métabolome de BordeauxVillenave d'Ornon, France; ^5^SupAgro, UMR BGPI Institut National de la Recherche Agronomique/CIRAD/SupAgro, Campus International de BaillarguetMontpellier, France; ^6^Institut National de la Recherche Agronomique, UMR BGPI Institut National de la Recherche Agronomique/CIRAD/SupAgro, Campus International de BaillarguetMontpellier, France

**Keywords:** defense, glutamine, fertilizer, *Magnaporthe oryzae*, nitrogen, pathogenicity, rice, effector

## Abstract

**Highlight**

Modifications in glutamine synthetase OsGS1-2 expression and fungal pathogenicity underlie nitrogen-induced susceptibility to rice blast.

Understanding why nitrogen fertilization increase the impact of many plant diseases is of major importance. The interaction between *Magnaporthe oryzae* and rice was used as a model for analyzing the molecular mechanisms underlying Nitrogen-Induced Susceptibility (NIS). We show that our experimental system in which nitrogen supply strongly affects rice blast susceptibility only slightly affects plant growth. In order to get insights into the mechanisms of NIS, we conducted a dual RNA-seq experiment on rice infected tissues under two nitrogen fertilization regimes. On the one hand, we show that enhanced susceptibility was visible despite an over-induction of defense gene expression by infection under high nitrogen regime. On the other hand, the fungus expressed to high levels effectors and pathogenicity-related genes in plants under high nitrogen regime. We propose that in plants supplied with elevated nitrogen fertilization, the observed enhanced induction of plant defense is over-passed by an increase in the expression of the fungal pathogenicity program, thus leading to enhanced susceptibility. Moreover, some rice genes implicated in nitrogen recycling were highly induced during NIS. We further demonstrate that the *OsGS1-2* glutamine synthetase gene enhances plant resistance to *M. oryzae* and abolishes NIS and pinpoint glutamine as a potential key nutrient during NIS.

## Introduction

Nitrogen fertilizers brought by the Green Revolution drastically increased yield but are also known to have increased the impact of many plant diseases (Dordas, 2008; Veresoglou et al., 2013; Fagard et al., 2014). The interaction between the blast fungus *Magnaporthe oryzae* and rice is no exception (Otani, 1959; Tanaka, 1961; Bonman, 1992; Sester et al., 2014). Given the economic impact of this disease as well as the fact that this interaction is a model for the analysis of cereal/fungal interactions (Dean et al., 2012), understanding the mechanisms by which nitrogen is inducing blast susceptibility is of major importance. In the field, there are several reasons that explain this phenomenon; for instance, tillering is increased by high nitrogen levels and as a consequence, the density of the canopy becomes more favorable to disease dispersion (Kuerschner et al., 1992). However, other mechanisms operating at the level of the plant can be proposed.

Nitrogen-Induced Susceptibility (NIS) is characterized by an increase in lesion number (Otani, 1959; Mukherjee et al., 2005; Talukder et al., 2005; Ballini et al., 2013) and a change in the type of lesions (Otani, 1959; Matsuyama and Dimond, 1973), suggesting that the cellular events associated with pathogen growth are affected. However, there has been no analysis at the cytological level of the events associated with this increase in lesion number or size. In particular, the frequency of penetration of the fungus, which could impact on lesion number, has not been established under high nitrogen fertilization.

At least four hypotheses can be proposed to explain the mechanisms of NIS. First, high nitrogen regime significantly impacts on plant growth (Makino, 2011) which in turn is critical for blast disease symptoms (Ribot et al., 2008; Vergne et al., 2010). This poses the almost unsolvable problem of distinguishing between the direct and indirect effects of nitrogen supply on disease resistance. In most of Otani's pioneer work (1959), significant effects on plants growth were associated with high nitrogen supply and the increase of blast symptoms. For instance, tillering was often significantly different between high and low nitrogen inputs. We have previously described an experimental system that can reproduce NIS of rice blast on leaf (Ballini et al., 2013). However, the effect of this protocol on plant growth has not been evaluated.

The second hypothesis postulates a modified trophic relationship to explain the increased fungal growth under high nitrogen levels. Amino acids are seen as a fuel and during infection a battle for fuel leads either to pathogen development/susceptibility or to nutrient remobilization/resistance (Bolton, 2009; Seifi et al., 2013). When total nitrogen was measured in parallel to rice blast symptoms, Otani (1959) found at best an increase of 22% between low and high nitrogen regimes. The severity of panicle blast of four genotypes was positively correlated to nitrogen concentrations in panicle tissues (Filippi and Prabhu, 1998). More recently, an increase of up to 78% total nitrogen was found by Talukder et al. (2005). For many pathogens, a significant increase in amino-acid content in the plant apoplasm and on the leaf surface may increase hyphal growth (Robinson, 1980). This is reminiscent of the observation that fungal growth is increased *in vitro* in the presence of high concentrations of nitrogen sources (Otani, 1959). *M. oryzae* is able to modulate plant nitrogen metabolism very early during the first phases of invasion, before 2 days post inoculation (dpi) (Parker et al., 2009; Fernandez and Wilson, 2012). Recently the *cks1* gene of *M. oryzae* required for cytokinin biosynthesis has been identified as also required for virulence (Chanclud et al., 2016). Interestingly the contents of glucose, aspartate and glutamate in infected plants were affected by the *cks1* mutation and high fertilization levels could restore *cks1* virulence. Thus, *M. oryzae* can modify the plant metabolism through the alteration of host hormone homeostasis.

Throughout the battle for fuel between plants and fungi, plant can resist using amino acids metabolism to remobilize nitrogen toward non-infected tissues and the production of defense reactions that are costly on energy (Bolton, 2009; Seifi et al., 2013). If nitrogen is a fuel for defense gene expression, one could expect that high nitrogen input would increase the expression of such genes. There are only very limited reports on the effects of nitrogen supply onto genes or pathways known to be required for disease resistance (Fagard et al., 2014; Suzuki et al., 2014). In rice, the few pieces of evidence on the effect of nitrogen on the expression of defense rather suggest that immunity is down-regulated by high nitrogen (Matsuyama and Dimond, 1973; Lian et al., 2006; Cai et al., 2012). It is noteworthy that in all these studies, the expression of plant defense has not been analyzed yet in *infected plants* grown under different nitrogen regimes. There is thus a major challenge in measuring the setting of immunity in plants that were differentially fertilized. Recently Kadotani et al. (2016) reported the induction of defense genes in leaves and roots after an amino acid treatment on the roots leading to an induced resistance to *M. oryzae*. This induced resistance was partially impaired in salicylic acid pathway mutants. Thus, a third hypothesis is that more than fuel, some key amino acids could directly act as regulators of the defense pathway (Kadotani et al., 2016). This is in accordance with the fact that several Arabidopsis mutants affected in the regulation of nitrogen metabolism have also been shown to be impaired for pathogen resistance (Camañes et al., 2012; Dechorgnat et al., 2012; Maekawa et al., 2012; Pastor et al., 2014). In rice *fd-gogat1* mutant genes confers resistance to *Xanthomonas oryzae* (Chen et al., 2016).

The fourth hypothesis states that, like in other pathosystems, fungal pathogenicity itself could be affected by *in planta* environment and in particular nutrient availability (Lau and Hamer, 1996; López-Berges et al., 2010; Saint-Macary et al., 2015). Indeed, the *in vitro* expression of several regulators of pathogenicity was shown to be induced by nitrogen starvation. For instance, the hydrophobin *MPG1* gene encoding a positive regulator of pathogenicity was induced *in vitro* by nitrogen starvation (Donofrio et al., 2006). Likewise, the transcription of several regulators of pathogenicity is under the control of regulators of nitrogen utilization (NPR1 and NPR2; Lau and Hamer, 1996). Moreover, it was shown that nitrogen starvation induced the expression of a large number of genes also found to be expressed during growth of the fungus in plant tissue (Talbot et al., 1997; Donofrio et al., 2006; Mathioni et al., 2011; Wang et al., 2011). Thus, nitrogen starvation *in vitro* enhances the expression of pathogenicity-related genes. This observation suggests that pathogenicity may require a nitrogen-poor environment for activation during colonization (Bolton and Thomma, 2008; Bolton, 2009; Wilson et al., 2012) and this is somehow a paradox with respect to the observation that high nitrogen regimes increase rice blast susceptibility (Ballini et al., 2013). The regulation of the expression of *M. oryzae* effector and pathogenicity genes in *infected plants* grown under different nitrogen regimes has not been yet documented and could help clarify the debate around the *in planta* relationship between nitrogen and pathogenicity (Solomon et al., 2003).

In this work, the growth of the rice blast fungus was examined before and after penetration to get more insight on the cellular events taking place during NIS. By conducting a dual RNA-seq experiment, we tried to identify the possible mechanisms of NIS. We show that the plant immunity is not reduced by nitrogen-fertilization. This led us to evaluate the effect of one glutamine synthetase gene in the rice blast interaction. Rice lines mutated for *OsGS1-2* were more resistant to rice blast and no longer showed enhancement of susceptibility under high nitrogen. We also provide evidence that the fungus is modifying its pathogenicity program in order to adjust to such new cellular environment.

## Materials and methods

### Plant growth and fertilization procedure

Plants were sown on Wednesdays in Neuhaus S soil in which poudzolane was added (2L/70L). Standard fertilization solution containing nitrogen source (75% ${\text{NO}}_{3}^{-}$/25% ${\text{NH}}_{4}^{+}$; 40 mg/L) was supplied every Monday for 3 weeks. Twenty six days after sowing, we supplied on Monday either a fertilization solution containing a nitrogen source (1N condition: 50% NH4^+^/50% NO3^−^; 40 mg/L), or the same solution without nitrogen source and corresponding to the 0N condition. This fourth fertilization was done 1 day before inoculation.

### Plant inoculation and disease assessment

Plants were inoculated with spore suspensions as described in Berruyer et al. (2003). For RNAseq experiments, we used GUY11 isolate as it is a reference for many *M. oryzae* experimental studies. For gene expression studies, we also used a mock treatment corresponding to the solution into which spores are re-suspended (i.e., 0.5% gelatin solution). Five to seven days after inoculation, the symptoms were scanned and the number of susceptible lesions were counted using ImageJ software (http://rsbweb.nih.gov/ij/) as described in Ballini et al. (2013).

### Quantification of total nitrogen and metabolic profiling

Plant tissues were harvested before and after fertilization. They were oven dried about 48 h at 65°C. Total nitrogen was analyzed in the samples by the Cirad analysis laboratory US49 in Montpellier (France). The content in nitrogen was established by a thermoelectric cell after burning in a furnace at 900°C under an oxygen flow according to the Dumas method. Amino acids and sugar contents were quantified as described in Gravot et al. (2010).

### RNA extraction and RT-PCR analysis

RNA extraction was performed as mentioned in Delteil et al. (2012). Quantitative PCR was performed using LC 480 SYBR Green I Master Mix (Roche, Basel, Switzerland) and a LightCycler 480 instrument (Roche). The amount of plant RNA in each sample was normalized using actin (Os03g50890) as internal control. PCR primers are provided in Supplementary Table 1.

### Tissue staining for confocal observation

Tissue staining was performed as described previously (Ballini et al., 2013). Observation were performed on LSM700 (ZEISS, http://www.zeiss.com) confocal microscope.

### RNA-seq data analysis

Total RNA was isolated with TRIzol (Invitrogen) from each sample according to manufacturer's instructions. It was treated with RNase-free DNase according to manufacturers's instructions (Promega) to remove residual DNA.

For RNA-seq, total RNA samples were sent to BGI Tech (Shenzhen, China) for sequencing. The libraries were sequenced as 101-bp paired-end reads using Illumina Hiseq2500 according to the manufacturer's instructions. Clean reads have been mapped using SOAP2 on reference genomes: Kasalath for rice and 70–15 for Magnaporthe. Annotated rice transcripts from Kasalath genome were downloaded from the TASUKE web-platform (Kumagai et al., 2013; Sakai et al., 2014). *M. oryzae* annotation datasets were downloaded from the Magnaporthe comparative Sequencing Project at the Broad Institute (assembly release 8; Chiapello et al., 2015).

The RNA sequencing depth allowed a good coverage of rice and *M. oryzae* genes. For one repetition, on average 15.9 × 10^6^ reads had a perfect match on Kasalath genome (non-inoculated or mock inoculated samples) and 46.1 × 10^6^ reads (inoculated samples). Up to 8,002 of the 31,507 Kasalath rice genes identified by the reads mapping had a low coverage (<5 reads) and were removed from the analysis in order to increase the statistical power. The sequencing depth allowed the detection of a large number of *M. oryzae* genes: on average 14,208 reads perfectly matched in an annotated gene in 0N samples and 37,864 reads in 1N samples. This difference is due to the higher amount of symptoms and thus of fungal RNA in the 1N condition. However, 7,324 genes had a low coverage and were removed in order to increase statistical power. FastQ files, reads counts and RPKM per gene are available on GEO database (GSE83219).

Differential expression between all repetitions was performed on DEB website (Yao and Yu, 2011) for each of the four conditions 0N mock inoculated, 0N Guy11 inoculated, 1N mock inoculated and 1N Guy11 inoculated. All the data were normalized together. For differentially expressed gene analysis we used DESeq with a minimum FDR of 5%. We considered as differentially expressed genes (DEGs) all genes that showed a significant difference in at least one of the four conditions. In order to conduct enrichment analysis we transferred when possible annotations from Nipponbare to Kasalath. We also used the Archipelago database referencing defense-related genes defense regulators (Vergne et al., 2008). Nitrogen pathway was annotated manually based on function annotation. Gene expressed after glutamine treatment were annotated based on GEO microarray GSE56770 (Kan et al., 2015). ABA responsive genes were collected based on literature (Gómez-Porras et al., 2007). Enrichment analysis in the different functions was tested against whole genome annotation with a Chi square test corrected by Bonferroni. GSEA was performed against whole genome on the rice array database (http://www.ricearray.org/).

For Differentially expressed gene analysis we used two different statistical analysis methods, namely DESeq and EdgeR with a minimum FDR of 5%. We considered as differentially expressed genes (DEGs) all genes that were significant in at least one of the statistical test. In order to perform enrichment analysis we did some complementary annotations. Gene coding for Carbohydrate-Active enZYmes (CAZY) were annotated based on CAZY.org database (Lombard et al., 2014). Pathogenicity genes were annotated based on literature. Small secreted proteins were annotated based on three criteria: (1) the presence of a signal peptide longer than 15 amino acids at the beginning of the protein, (2) the absence of a transmembrane (TM) domain or a TM domain in the first 10 aa and (3) a protein size smaller than 250 aa. Signal peptide was detected using SignalP program (Petersen et al., 2011). Transmembrane domains was detected using TMHMM program (Krogh et al., 2001). Enrichment analyses was performed on Fungidb database (Stajich et al., 2012) and for the selected functions the frequency was tested against the whole genome annotation with a Chi square test corrected by Bonferroni.

## Results

### Growth and nutrients during nitrogen-induced susceptibility

We have previously described a laboratory protocol inducing NIS in the Kasalath but not in the Nipponbare variety after inoculation with *M. oryzae* (Ballini et al., 2013). Plants were grown during 4 weeks and 1 day before inoculation they are separated in two sets: fertilized without nitrogen (0N: all macro and micro-elements except N) or full fertilization (1N). An increase of leaf elongation was visible at 2 dpi in Kasalath (NIS) but not Nipponbare (no NIS) (Supplementary Figure 1). This increase was moderate and almost undetectable at the end of the experiment, when disease symptoms were developed (5 dpi). Thus, our experimental system minimizes the impact on plant growth.

Several evidences suggest a correlation between total nitrogen content and blast susceptibility (Otani, 1959; Talukder et al., 2005). Therefore, we measured total nitrogen content in rice leaves before and after infection in plants grown under 0N and 1N regimes (Supplementary Figures 2A,B). Total nitrogen content was not affected in Nipponbare before or after inoculation. However, in Kasalath total nitrogen content was significantly higher by up to 8.5% under the high nitrogen regime at 2 dpi. Thus, a slight increase in total nitrogen content is observed during the fungal invasive phase in NIS. To investigate in more detail the changes in nutrient contents, total amino acids and three sugars were quantified in rice leaves in three independent experiments (Supplementary Figures 2C–F). Before inoculation, no significant change could be observed between 0N and 1N samples in Kasalath or Nipponbare (Supplementary Figures 2C,D). In Nipponbare no significant change could be observed at 2 dpi although there was a tendency toward less metabolites detected in 1N samples. On the contrary, at 2 dpi a significant difference was observed in Kasalath for glutamine and alanine in mock inoculated plants but not in inoculated plants. The overall tendency in Kasalath for the other metabolites was an increase in 1N samples. However, further analysis will be necessary to establish if the changes in glutamine contents in *M. oyzae* inoculated samples could be causal for NIS.

### Nitrogen fertilization increases susceptibility to a large set of *M. oryzae* strains

**Figure 1:**
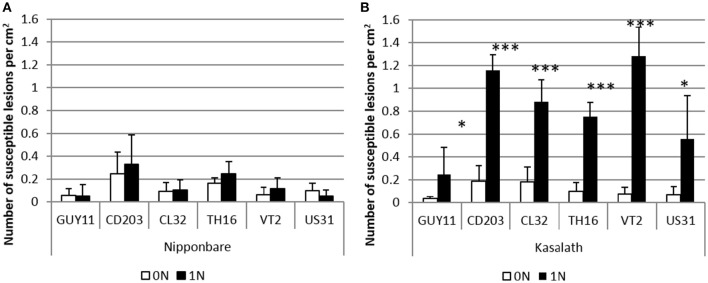
**Effect of nitrogen supply on rice blast disease in Nipponbare and Kasalath genotypes**. Low or high nitrogen fertilization (0N, 1N; see Section Materials and Methods) was applied to rice 1 day before inoculation with different isolates of *M. oryzae*. The number of susceptible lesions was measured with six different isolates on Nipponbare **(A)** and Kasalath **(B)**. The mean and sd of three replicates is shown. This experiment was repeated twice and one representative replicate is shown. ^*^Student test; *P* < 0.05; ^***^Student test; *P* < 0.001.

In our experimental system, disease symptoms on Kasalath plants, as measured by the number of susceptible lesions, were significantly increased with high levels of nitrogen fertilization (Figure 1, Supplementary Figure 3A). We reproducibly observed from 2 to 6 times more lesions for the Guy11 isolate and also an increase of lesion size, up to 2.2-fold (Supplementary Figure 3B). This is consistent with what was found by Otani (1959). In contrast, disease severity in the Nipponbare genotype against the Guy11 isolate was not affected (Figure 1, Supplementary Figures 3, 4).

Our NIS system had not been tested in other rice/isolate combinations. To evaluate if the observed effects of nitrogen supply were specific to the Guy11 isolate, we further tested five virulent rice blast isolates (Figure 1, Supplementary Figure 4). Nitrogen supply did not trigger any significant increase of susceptibility in Nipponbare. By contrast, disease severity in Kasalath was higher with the six isolates of *M. oryzae* tested after nitrogen supply (Figure 1, Supplementary Figure 4). The increase in lesion number triggered by nitrogen supply varied from a maximum of 17-fold with the VT2 isolate and a minimum of 5-fold with the CL32 isolate. This protocol also induced enhanced susceptibility in a large range of rice genotypes (Ballini et al., 2013). Thus, our experimental NIS protocol has a minimal impact on plant development and allows studying the mechanisms of NIS in a panel of rice variety/*M. oryzae* isolates with differential response to nitrogen.

### Cytological events associated with nitrogen-induced susceptibility

**Figure 2:**
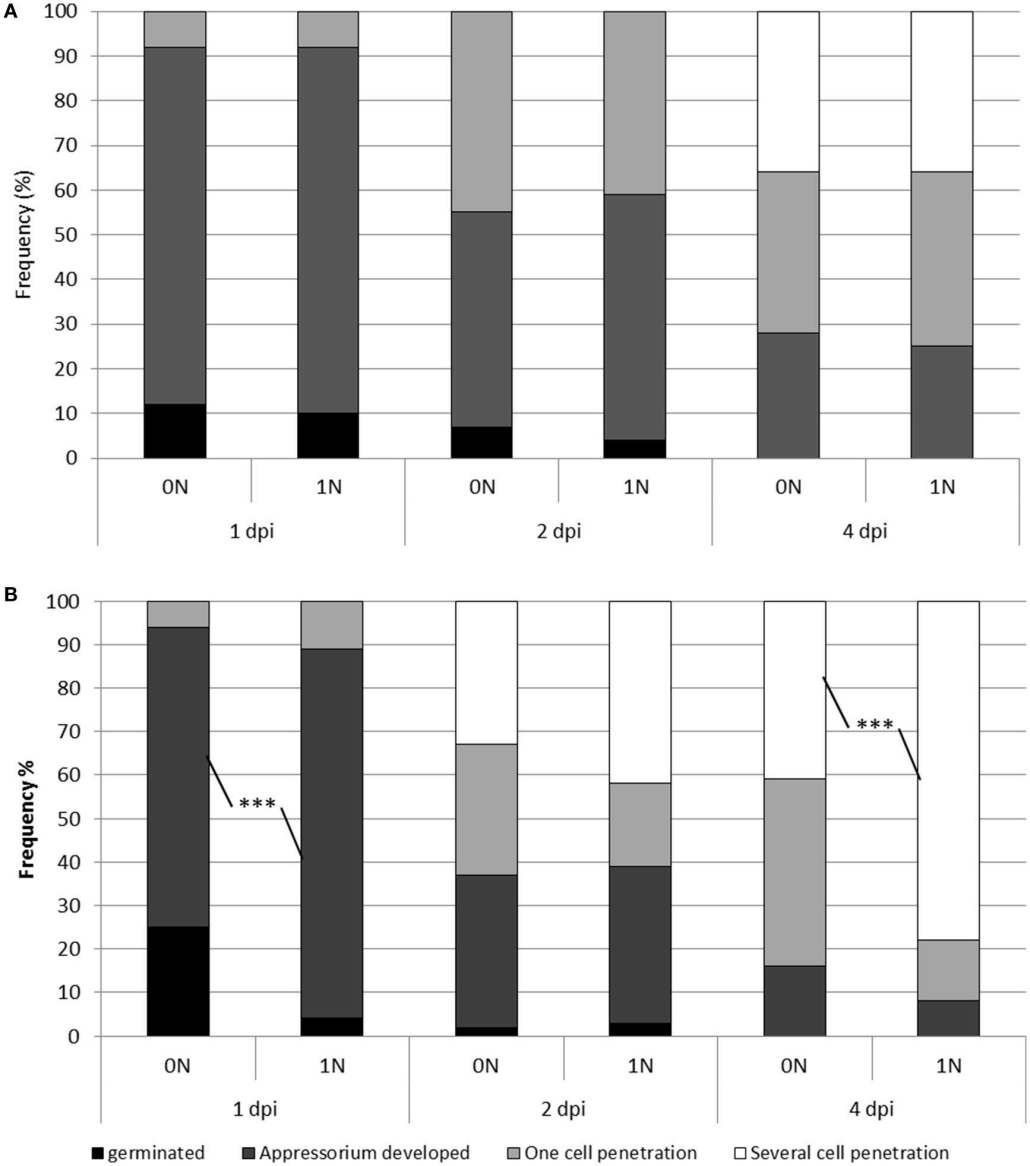
**Penetration and growth of *M. oryzae* in rice plants under different nitrogen regimes**. Low or high nitrogen fertilization (0N and 1N; see Section Materials and Methods) was applied to Nipponbare **(A)** and Kasalath **(B)** plants which were subsequently inoculated with the Guy11 isolate. At the indicated time after inoculation (1, 2, and 4 dpi), the developmental stage of the fungus was evaluated. Four types of situations were counted: a spore that germinated but did not develop an appressorium (black), a spore with a developed appressorium (dark gray), sites where the fungus had penetrated one cell (light gray) and sites where the fungus had penetrated several cells (white). For each time x treatment combination, a total of 100 events were counted. This experiment was repeated three times and one representative experiment is shown. A Chi square test was used to compare the different percentages (see text). ^***^P < 0.001.

Up to now, there has been no evaluation of the effects of nitrogen supply on the growth of the blast fungus before and after penetration in the rice leaf. We monitored the growth of the isolate Guy11 in Kasalath and Nipponbare plants at different time points after inoculation (1, 2, and 4 dpi) and under the 0N and 1N conditions. As shown in Figure 2A, we observed no difference in Nipponbare between the 0N and 1N conditions. This is consistent with the absence of increased susceptibility in this cultivar (Figure 1). In contrast, several significant differences were found in Kasalath plants (Figure 2B). First at 1 dpi, 85% of the spores developed its specialized penetration cell, appressorium, under 1N conditions compared to 70% under 0N conditions. Thus, the fungus seems to grow faster on the leaf surface of plants that were supplied with nitrogen. This difference in fungal growth is no longer observed at 2 dpi as the fungus has penetrated in 60–65% of the cases, irrelevantly of the nitrogen regime. Thus, the final penetration efficiency does not seem to be affected by nitrogen supply. Finally, at 4 dpi, before the first symptoms appear on the leaf surface, we could measure a significant difference in the percentage of cells that each individual colony had invaded. Whereas, 45% of the fungal colonies invaded several cells under the 0N condition, more than 80% could be observed under the 1N condition. Therefore, the growth of the blast fungus inside the plant cells is higher after nitrogen supply. Based on these results we decided to further examine the molecular changes occurring at 2 dpi, when fungal growth does not seem to be significantly affected and before the strong changes observed later on at 4 dpi.

### Transcriptomic changes during NIS

In order to investigate the transcriptional regulation during NIS in Kasalath inoculated with Guy11, we decided to conduct a dual RNA-seq experiment. In this way we were therefore able to follow both rice and *M. oryzae* gene expression before (only for rice) and 2 days after inoculation. The data are available on GEO database (GSE83219).

At 0 dpi RNA-seq analysis on rice genes allowed the detection of 243 differentially expressed genes (DEG): 65 are more expressed in 1N and, 178 are less expressed in 1N (Supplementary Table 2). The 65 highly expressed genes are enriched in defense-responsive genes present in the rice defense database (Vergne et al., 2008). Thus, this analysis shows that there is no massive modification of rice transcription 1 day after nitrogen supply in our condition, although some defense regulators may be induced.

**Figure 3:**
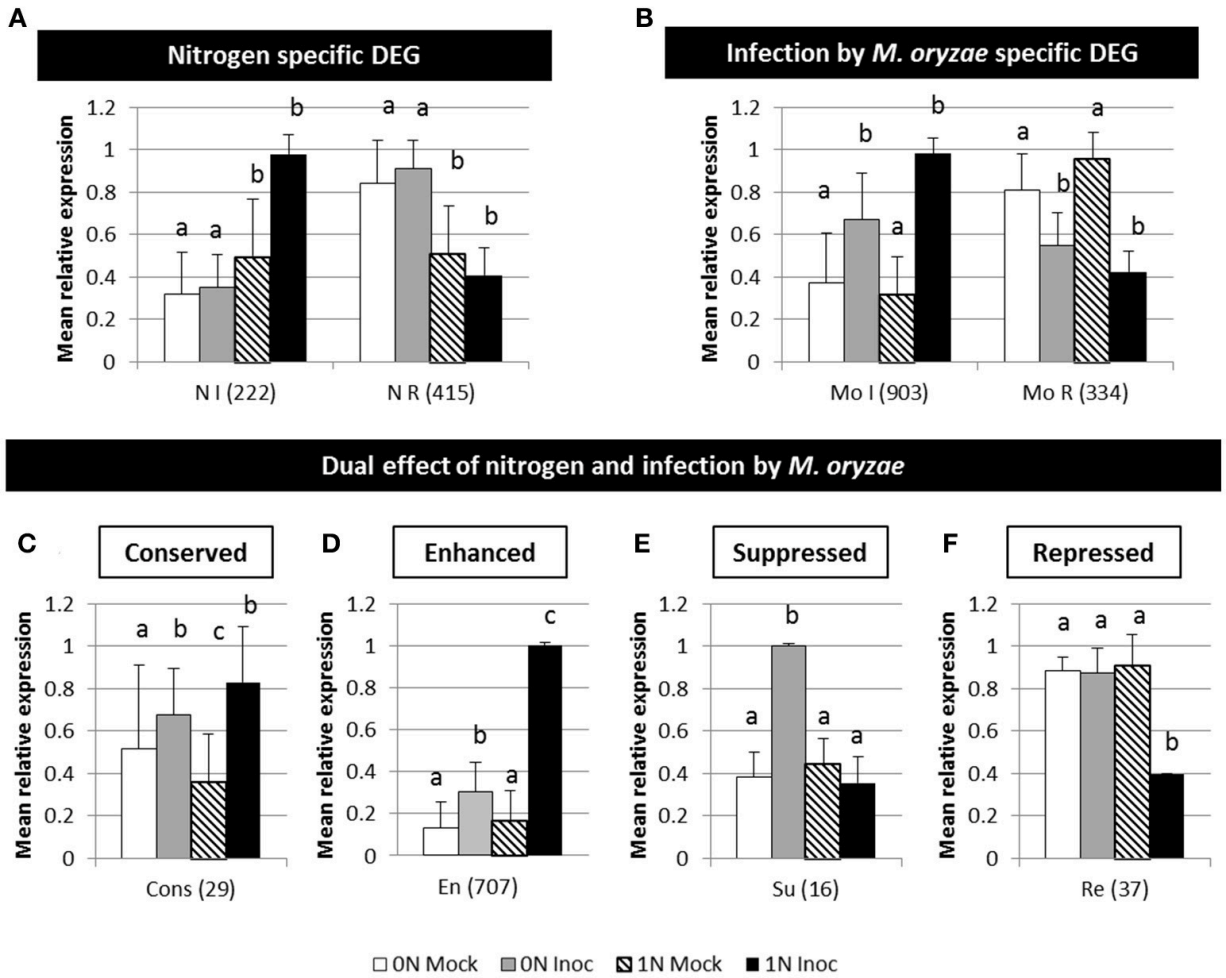
**Major type of patterns of rice differentially expressed genes during infection under different nitrogen fertilization. (A)** Nitrogen specific genes: genes differentially expressed between 0N and 1N treatment but not differentially expressed during infection. **(B)** Gene expression profile specific of infection by *M. oryzae*. **(C)** Gene expression level conserved between 0N and 1N during infection. **(D)** Enhanced response to inoculation in the 1N condition. **(E)** Suppression of response to inoculation in 1N condition. **(F)** Repression by infection only in the 1N condition. Four different conditions were compared with Deseq2 analysis: 0N fertilized and mock inoculated (white), 0N fertilized and Guy11 inoculated (light gray), 1N fertilized and mock inoculated (black dash) and 1N fertilized and Guy11 inoculated (black). The six global patterns indicated here represent the mean and sd expression values of the considered group of genes, as identified by analysis of the *p*-value obtained for each comparison with Deseq2. For each histogram, a given letter identifies similar values. The values between parentheses represent the number of genes in each category. I, induced; R, repressed; N, nitrogen; Mo, *Magnaporthe oryzae*; Cons, conserved; En, enhanced; Su, suppressed; Re, repressed. Kasalath plants were fertilized with low (0N) and high (1N) nitrogen fertilization 1 day before inoculation. Plants were inoculated with the Guy11 isolate or a mock solution. RNA were extracted 2 days after infection and analyzed using RNA-seq (see Section Materials and Methods).

At 2 dpi, differentially expressed genes were detected by comparing four different possible situations: No nitrogen and mock inoculation (0N Mock), nitrogen-fertilized and mock inoculation (1N Mock), no nitrogen and Guy11 isolate inoculation (0N Inoc) and last nitrogen-fertilized and Guy11 isolate inoculation (1N Inoc; Figure 3, Supplementary Table 3). We considered a gene as differentially expressed (DEG) when DESeq analysis gave an adjusted *P* < 0.05. Previous studies indicate that the transcriptome of combined treatments is poorly predicted by the transcriptome of single treatments (Rasmussen et al., 2013; Suzuki et al., 2014) and we made a similar observation. For instance, a total of 2,711 DEGs were identified with DESeq analysis between “1N Inoc” condition and “0N Mock” condition while only 1,848 of these genes (68%) could be predicted when analyzing the four conditions separately.

Using our statistical analysis on the four conditions separately, we could identify a total of 2,665 DEGs in six groups (Figure 3). We compared all group of genes (list of genes in Supplementary Table 3) showing similar expression pattern with available known groups of genes involved in disease resistance (Vergne et al., 2008 and updated versions) or in nitrogen/glutamine response (GEO database; GSE56770) and enrichment values are given in Supplementary Table 4.

The first group represents 637 nitrogen-specific DEGs that are genes differentially expressed between 0N and 1N conditions independently of infection: 222 are induced and 415 are repressed by nitrogen (Figure 3A). No detectable enrichment in gene related to nitrogen pathway was observed in this experiment, which suggests that transcriptional changes following fertilization may have happened earlier than 2 dpi to increase the observed nitrogen content (Supplementary Figure 2). The second group represents 1,237 DEGs specific to infection by *M. oryzae* (Figure 3B) as they are differentially expressed during infection independently of the nitrogen treatment: 903 are induced and 334 are repressed by infection. As expected, this group of DEGs is enriched in defense-related genes like PR proteins. While these first two groups represent classical nitrogen-related and infection-related situations, the four remaining groups (representing 789 DEGs; Figures 3C–F) contain genes that are differentially expressed by both nitrogen *and* infection conditions.

The third group gathers 29 DEGs genes whose expression is normally modified by nitrogen or infection but is globally conserved between 0N and 1N during infection (Figure 3C). Infection may prioritize the expression of these genes despite a negative cross talk with nitrogen regulation. The final level of expression of these genes is conserved at 2 dpi whatever the fertilization used. This group is enriched in WRKY transcription factors and response to biotic stress.

The fourth group represents 707 DEGs differentially expressed during infection with an amplified response to inoculation in 1N condition (Figure 3D). This family is enriched in PR proteins, defense regulators, phytoalexin pathway, WRKY transcription factors and response to biotic stress. Several known positive and negative defense regulators are part of this group like some regulators of transcription (OsWRKY62, OsWRKY28, OsNAC4, OsbHLH148, OsSpl7, NH1, and OsNPR4), an allene oxide synthase involved in JA biosynthesis (OsAOS2), a salicylic acid 3-hydroxylase (OsS3H) and several kinases (OsBSR1, OsBIDK1, OsMPK5). Thus, the transcription of defense-related genes and regulators is amplified in 1N condition at 2 dpi although plants are finally more susceptible (see Discussion). Interestingly this group is also enriched in glutamine responsive genes like the *OsGS1-2* gene coding for a glutamine synthetase (Supplementary Figure 5; Kan et al., 2015). This is in accordance with previous results showing that *OsGS1-2* is expressed at a lower level in rice roots grown with low nitrogen (Funayama et al., 2013; Yang et al., 2015). This observation was followed-up by the analysis of a rice *gs1-2* mutant during NIS (see below).

The fifth group (Figure 3E) is composed of 16 genes which response to inoculation is suppressed in 1N condition. The sixth group is composed of 37 genes (Figure 3F) that are repressed during infection under the 1N condition only. These two last groups potentially represent functions that are impacted negatively by high nitrogen and that may otherwise favor resistance; however, there was no massive enrichment of genes involved in disease resistance in this group. Instead we found several genes that are related to senescence like *OsNAP* and *OsTZF1*. The OsNAP transcription factor, whose induction by inoculation under 0N is abolished in 1N (Figure 3E), has a pivotal role in senescence regulation (Liang et al., 2014) and jasmonic acid biosynthesis (Zhou et al., 2013). Similarly, the OsTZF1 transcription factor that is repressed in infected plants under 1N but not 0N (Figure 3F) is known to inhibit senescence and to induce defense-related genes and HR-like lesions (Jan et al., 2013). Thus, the fifth and sixth DEG groups gather genes that could be implicated in NIS regulation based on their expression profiles. As expected, genes involved in nitrogen assimilation and remobilization are among these groups (Supplementary Table 3) and we followed-up the hypothesis that such genes are key determinant of NIS.

### Glutamate metabolism is only slightly modified during NIS

**Figure 4:**
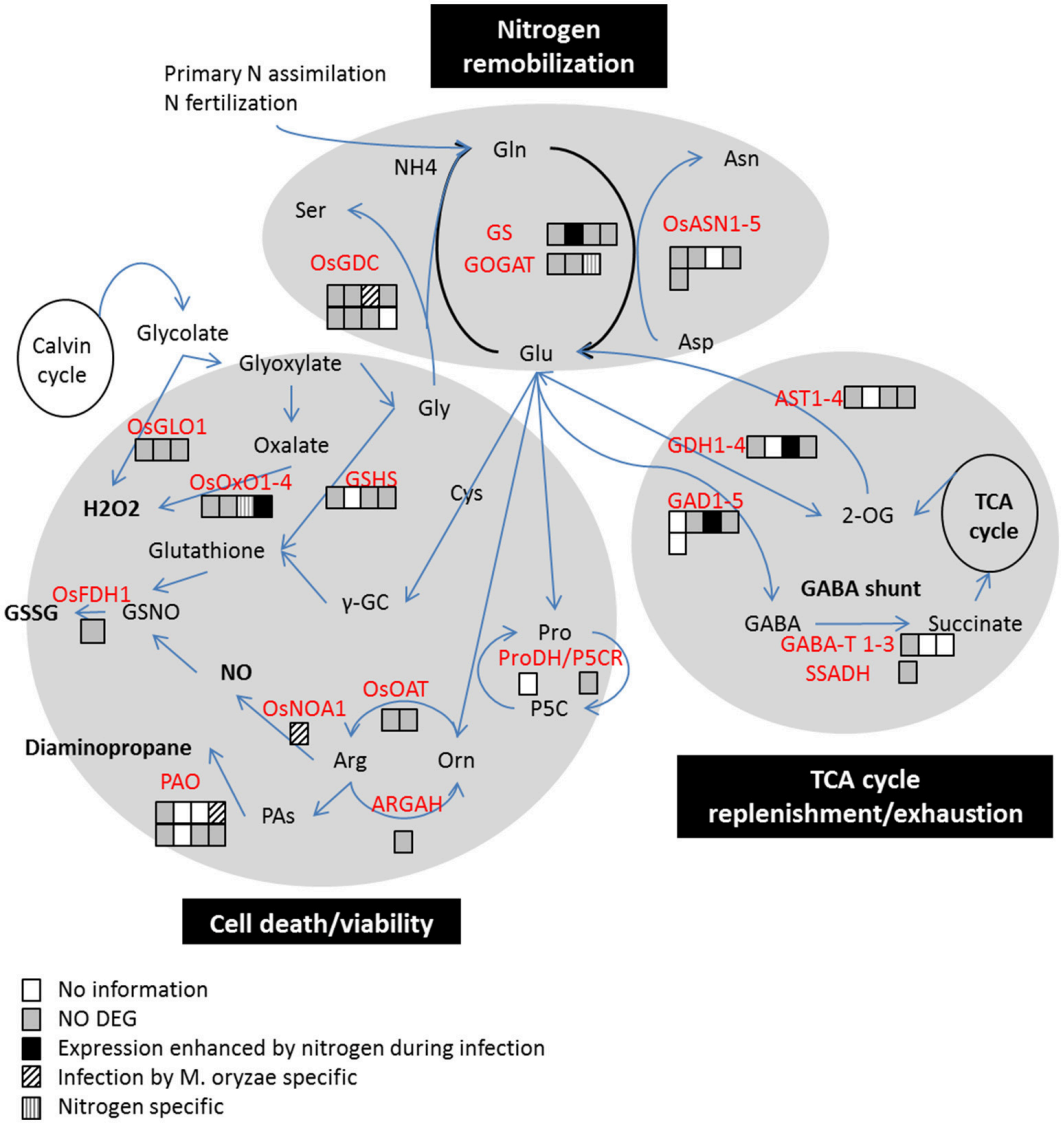
**Three major pathways potentially in conflict between the host metabolism and pathogen virulence**. Adapted from Seifi et al. (2013). Differential expression in RNA-seq at 2 dpi are represented by colored squares for each gene coding for key enzyme. The patterns refer to categories described in Figure 3. The main enzymes implicated in the nitrogen remobilization away from infected tissues are GS, Gln Synthetase; GOGAT, Glutamate synthase; GDC, glycine decarboxylase; ASN, Asn synthetase. The main enzymes implicated in the cell death pathway linked to GS/GOGAT are ProDH, Proline dehydrogenase; ARGAH, P5C reductase, arginase; OAT, Ornithine carbamoyl transferase; NOS, nitric oxide synthase; PAO, polyamine oxidase; GSHS, glutathione synthetase; OsOXO1-4, Oxalate oxidases; OsGLO1, glycolate oxidase. The main enzymes for TCA cycle and GABA-shunt pathways are AST, Asp transaminase; GDH, glutamate dehydrogenase; GAD, glutamate decarboxylase; GABA-T, and SSADH.

Glutamate metabolism is not only crucial for amino acid metabolism but it has been reported as a key player in defense (Seifi et al., 2013). In their review Seifi et al. have reported that glutamate metabolism functions in opposite ways: fueling plant defense or diverted by the pathogen to facilitate infection. They identified three major pathways that may lead to resistance or susceptibility: Nitrogen remobilization away from infected tissues, TCA cycle replenishment and cell death. We used this review to identify the genes associated with these three defense strategies that were identified in our RNA-seq data set using annotation (Figure 4) and looked if these pathways were affected in infected plants under high nitrogen. We identified 18 genes implicated in nitrogen assimilation in our dataset: 15 are not differentially expressed in none of the four considered conditions at 2dpi. *OsGS1-2* is the only gene whose response to *M. oryzae* is amplified by nitrogen treatment (Figure 3D). This expression pattern is not correlated with the detected level of glutamine in the leaves at 2 dpi (Supplementary Figure 2F). When comparing plants grown under 0N and 1N regimes, an increase in glutamine level was observed in mock inoculated samples but not in *M. oryzae* inoculated samples. In the same experiment used for RNAseq analysis, *OsGS1-2* expression increased at a higher lever under 1N regime than under 0N regime. OsNADH-GOGAT2 is nitrogen specific induced (Figure 3A) and a GDC (Os06g45670) is induced by *M. oryzae* at a level that is not significantly different in both N treatments. We identified 21 genes associated to several metabolism process that possibly link the cell death induction to GS/GOGAT pathway in our dataset: 17 are not differentially expressed in any of the conditions. *OsNOA1* is repressed and *OsPAO4* is induced during infection in both nitrogen regimes. *OsOXO4* is the only gene whose transcriptional response to *M. oryzae* is amplified by high nitrogen. However, the role of *OsOX4* in rice blast resistance is not clear (Zhang et al., 2013; Li et al., 2015). Finally, we identified in our datasets 11 genes coding for enzymes potentially involved in the TCA cycle replenishment or GABA shunt, 9 of which are not differentially expressed. *OsGDH3* and *OsGAD3* have an amplified response to *M. oryzae* in presence of nitrogen. *OsGAD3* and *OsGDH3* may be controlled by the pathogen in order to boost TCA cycle and compensate the drain of nutrient away of the infected tissues. In N and P deprived environments, *OsGDH3* is induced in rice shoots probably to boost nitrogen recycling (Qiu et al., 2009). Thus, we did not detect a massive transcriptional reprogramming of the three pathways identified by Seifi et al. (2013) during infection under high nitrogen. However, the modification of the regulation of some of the key enzymes by infection could explain NIS and to test this hypothesis, we analyzed the role of *OsGS1-2* in defense.

### Glutamine synthetase and NIS

**Figure 5:**
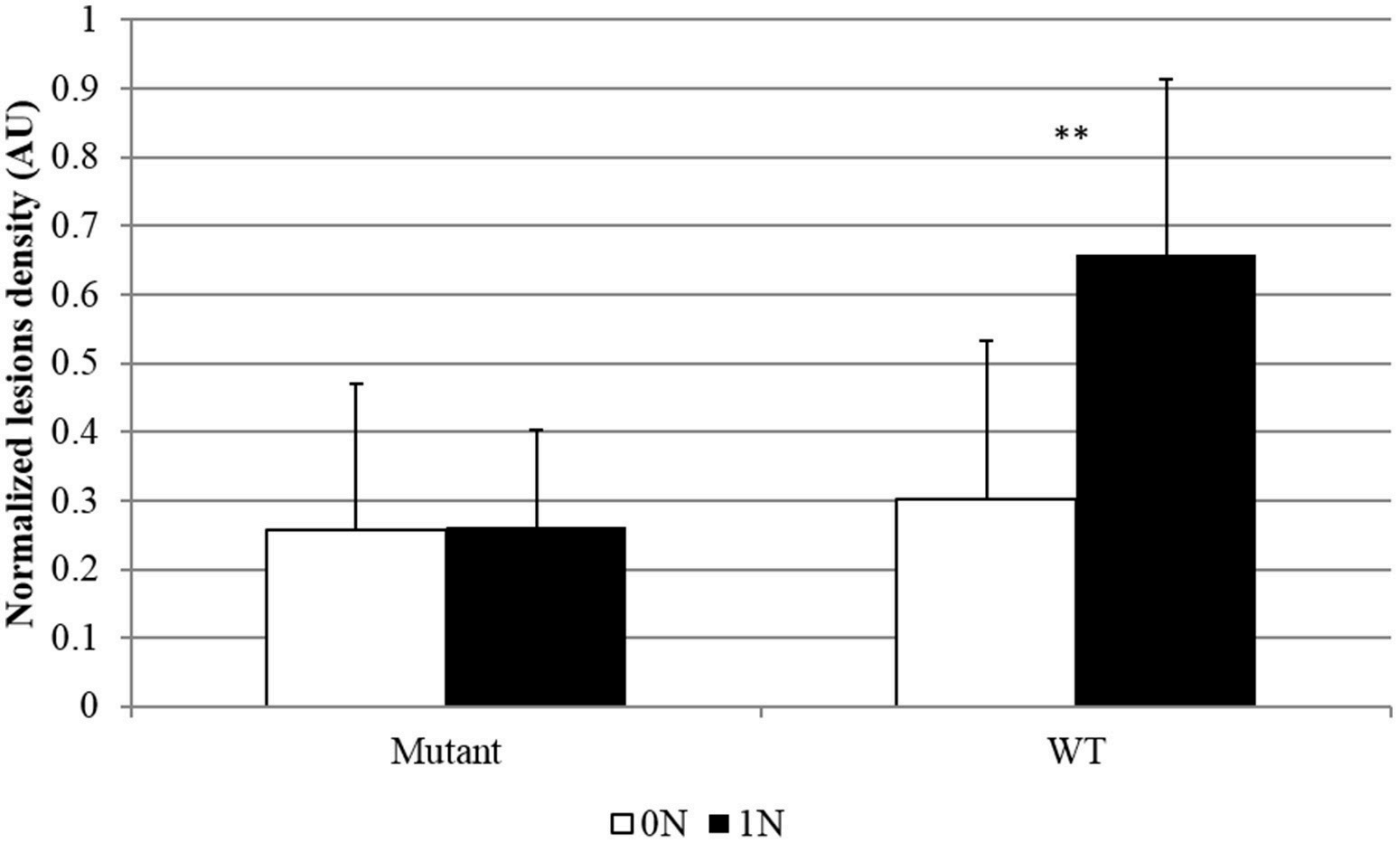
**Rice blast symptoms in OsGS1-2 mutant and wild type lines**. Mutants and corresponding Dongjin WT lines not containing the T-DNA were obtained from the T-DNA insertion line 1B10506 mutated in the *OsGS1-2* gene (Only Heterozygous mutants-He- were found; see Supplementary Figure 6). Symptoms caused by *M. oryzae* (isolate Guy11) were estimated by counting the number of susceptible lesions 5 days after inoculation on eight different replicates. Different doses of nitrogen fertilization (0N, 1N; see Section Materials and Methods) were supplied to rice 1 day before inoculation. This experiment was repeated three times and 12 independent replicates were obtained. Data were normalized between the two replicates using the symptoms obtained for the most susceptible plants in each replicates. ^**^Student test; *p* < 0.01.

The GS enzyme catalyzes the conversion of glutamate to glutamine using ${\text{NH}}_{4}^{+}$. In rice there are three genes coding for isoenzymes of cytosolic glutamine synthetase (*OsGS1-1, OsGS1-2*, and *OsGS1-3*). One of these genes appears as a good candidate for NIS mechanisms investigation: *OsGS1-2*, which was specifically induced in infected plants under high nitrogen (Supplementary Figure 5). This gene, is important in the primary assimilation of ${\text{NH}}_{4}^{+}$ taken up by rice roots (Yamaya and Kusano, 2014). The knock-out *gs1-2* mutants showed reductions in the content of glutamine, glutamate, asparagine, and aspartate, and an increase in free ${\text{NH}}_{4}^{+}$ ions in the roots, shoots and xylem sap (Funayama et al., 2013). We obtained a knock-out mutant in the *OsGS1-2* gene using a line where a T-DNA is inserted at the end of the coding region (Supplementary Figure 6A). The expression level of *OsGS1-2* was strongly reduced (Supplementary Figure 6B) while plant growth was not affected in this *gs1-2* mutant. Under our growth conditions, we were able to detect a slight decrease in fructose, glucose and some amino acids in the shoots of the *gs1-2* mutant compared to the wild type (Supplementary Figure 7). Infection assays with *M. oryzae* of wild-type and *gs1-2* mutants were done under low and high nitrogen. We observed that the *gs1-2* mutants were more resistant than wild-type plants (Figure 5). More importantly, while wild-type plants showed a typical NIS phenotype, the *gs1-2* mutants did not show an increased susceptibility under high nitrogen fertilization.

### High nitrogen fertilization and *M. oryzae* pathogenicity genes

**Figure 6:**
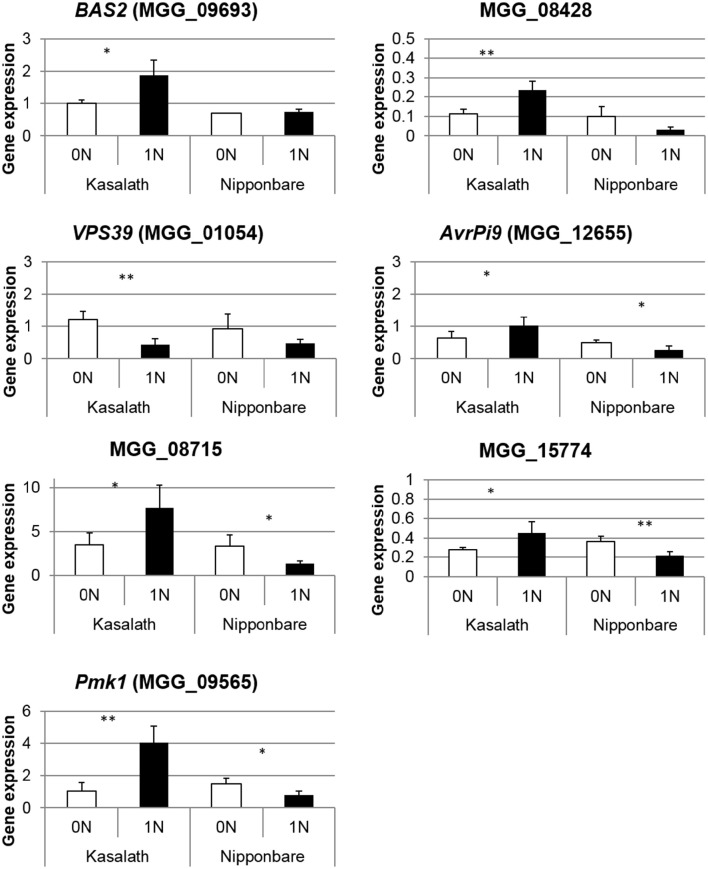
**Validation of RNA-seq for *M. oryzae* pathogenicity genes by quantitative RT-PCR**. The expression of *M. oryzae* pathogenicity genes was measured in a biological replicate of the RNA-seq experiment. *M. oryzae* was inoculated on two varieties (Kasalath and Nipponbare) and only Kasalath showed NIS phenotype as in Figure 1. Gene expression was normalized with fungal actin (MGG_03982). Student test was used to compare 0N and 1N conditions. ^*^P < 0.05, ^**^P < 0.01. Only the seven significant genes are represented and other genes are shown in Supplementary Figure 8.

The dual RNA-seq experiment allowed us to follow fungal gene expression *in planta* at a time (2 dpi) where we did not measure any difference in fungal growth inside tissues from rice fed with or without nitrogen (Figure 2). Of the 60 million reads sequenced in each of the six experiments at 2 dpi, ~26,000 perfectly matched *M. oryzae* genes and not rice. We decided to compare the expression between the two nitrogen treatments for genes with a minimum coverage of 5 reads (3428 genes out of *M. oryzae* 10,752 genes detected; Supplementary Table 5). Overall, this set of 3,428 genes expressed *in planta* at 2 dpi is significantly enriched in genes coding for SSP (small secreted protein: 293 genes; 8.5 against 6.2% expected from genomic data) and known pathogenicity genes (2.1 against 1.3%) but not in cell-wall degrading enzymes (CAZY) (4.2 against 3.8%) (Supplementary Table 6), consistent with previous results of *M. oryzae* expression *in planta* (Matsumura et al., 2003; Mosquera et al., 2009; Kim et al., 2010; Mathioni et al., 2011; Kawahara et al., 2012). Only a few genes (24) were significantly differentially expressed between our 0N and 1N conditions and most had unknown functions (Supplementary Table 7). In order to get further insight of the global trends of gene expression in *M. oryzae*, we analyzed the global and not the individual fold change of some functional categories amongst the 3,428 investigated genes. In this way, we could detect tendencies in the pattern of gene expression of specific biological or molecular functions. Of the 293 genes coding for SSPs, 131 were more expressed in 1N condition (Log_2_ fold change >0,7) whereas only 32 were less expressed in 1N condition (Log_2_ fold change < −0,7) (Supplementary Table 5). This tendency was not observed for other pathogenicity genes, vesicle trafficking components, transcription factors or CAZY enzymes (Supplementary Table 5). In order to validate this tendency, we tested the expression of a set of 19 genes coding for SSPs and other pathogenicity factors by Q-RT PCR in a biological replicate of the RNA-seq experiment (Kasalath plants showing NIS). Seven of these genes were indeed differentially expressed as expected from RNA-seq data (Figure 6): *Pmk1* (Thines et al., 2000), *Bas2* (Mosquera et al., 2009), *AvrPi9* (Wu et al., 2015), and three putative effectors (MGG_08715, MGG_15774 and MGG_08428) were more expressed whereas VPS39 (Ramanujam et al., 2013) was less expressed in 1N conditions. Interestingly, all these genes were less expressed in 1N condition in Nipponbare (where NIS was not observed). The 12 other genes tested were not differentially expressed in either variety (Supplementary Figure 8). This data suggests that *M. oryzae* can sense the physiological status of the plant and potentially adapt its pathogenicity strategy by delivering a higher amount of effectors in high nitrogen conditions.

## Discussion

From bibliographical analysis, at least four non-exclusive hypotheses can be proposed to explain Nitrogen-induced Susceptibility: (1) an indirect effect via plant growth, (2) an increase in nutrient availability for the fungus, (3) a regulation of plant immunity by nutrients like amino acids and (4) a direct, positive regulation of fungal pathogenicity functions by nutrient availability. Using the interaction between rice and the blast fungus *M. oryzae*, we provide some elements to these different hypotheses and propose a model for the possible molecular mechanisms triggering NIS.

### Nitrogen fertilization induces small physiological and morphological changes but strongly impacts blast susceptibility

Rice blast disease is highly dependent on the developmental stage of the plant (Vergne et al., 2010) but several results presented here with our NIS protocol argue against the possibility that NIS is an indirect effect of nitrogen on plant development. In our system, there was only a limited modification of plant growth after infection (Supplementary Figure 1). Our way of applying nitrogen for triggering NIS did not strongly affect plant physiology, as shown by total nitrogen and metabolome measurements (Supplementary Figure 2). Some amino acids like glutamine and alanine significantly but slightly increased in plant supplied with nitrogen and a similar trend could be observed for other metabolites. Plant transcriptome was also only moderately affected by nitrogen fertilization, as shown by RNA-seq data before infection or in mock treated samples at 2 dpi (Supplementary Tables 2, 3). Indeed, while infection alone affected more than 1,237 genes at 2 dpi, nitrogen alone only affected 243 genes before infection (Supplementary Table 2) and 639 genes at 2 dpi (Figure 3). Thus, based on physiological, metabolomics and transcriptomic results, we conclude that our NIS protocol is only slightly affecting plant growth and plant physiological processes in the absence of infection whereas it strongly impacts on disease severity. Further investigation is required to confirm if the slight increase in some metabolites (e.g., glutamine) could be causal of NIS.

### Nitrogen fertilization has a complex impact on defense expression

Although a link between amino acids metabolism and defense response has been demonstrated several times, a remaining question is how nitrogen fertilization affects defense. Nitrogen fertilization and subsequent modifications of amino acid content could play a role in fueling the defense response (Masclaux-Daubresse et al., 2006; Bolton, 2009; Nussbaumer et al., 2015; Park et al., 2015) or in regulating key defense regulators (Rojas et al., 2014). Similarly to previous results, we observed in our dataset that defense-related genes (e.g., *PR* genes and genes involved in the biosynthesis of the antimicrobial secondary metabolites) are more expressed during infection under high nitrogen fertilization (Figure 3). However, our results also show that there could be a complex regulation of defense in nitrogen fed plants. Indeed despite the amplification observed for *PR* genes and eight positive regulators of defense, several negative regulator of defense were also strongly induced by high nitrogen (*OsWRKY62, OsWRKY28, OsMAPK5, OsNPR4, OsS3H*). Thus, we cannot exclude the possibility that among the many types of defense regulators induced by high fertilization, some negative components may be critical for increasing susceptibility. However, if this was the case, one would expect end-executers like *PR* genes to be down-regulated, which is not the case. More subtle cases could also explain how high nitrogen may impact on susceptibility, for instance the case of the induction of *OsNAP* gene by infection found to be abolished by high nitrogen (Figure 3E). OsNAP negatively regulates the biosynthesis of abscisic acid (ABA; Liang et al., 2014) and positively regulates the biosynthesis of jasmonic acid (Zhou et al., 2013). Since ABA and jasmonic acid are respectively a negative (Jiang et al., 2010) and a positive (Shimizu et al., 2013) regulator of rice resistance to blast, the induction of OsNAP could also participate indirectly to defense. The absence of induction of *OsNAP* by infection under high nitrogen would prevent the proper setting of immunity. Thus, based on our results, we propose that the increased susceptibility in nitrogen-treated plant *cannot* be explained by a general down-regulation of the defense pathway.

### Nitrogen fertilization likely fuels *M. oryzae*

The idea that nitrogen fertilization increases nutrient availability and thus pathogen growth is supported by our results. First, it seems that fungal growth may increase on the plant surface under high nitrogen (Figure 2) at a time where there was no obvious down-regulation of plant defense (Supplementary Table 2). In other systems (for review see Robinson, 1980), it has been observed that germination and growth of the fungus on the plant surface was increased by high nitrogen levels. This suggested that the pathogen can sense on the leaf surface some metabolic modifications of its host consecutive to nitrogen fertilization, potentially amino acid levels. Fungal growth was also increased in infected plant tissues at 4 dpi (Figure 2). Similar results were observed for colonies of *Blumeria graminis* f.sp. *hordei* grown on barley treated with high nitrogen fertilization (Newton and Guy, 1998). Second, the *gs1-2* mutants, which showed a reduction in sugars and amino acids in leaf tissues before infection, were more resistant to *M. oryzae* (Supplementary Figure 7). This decrease in some sugars and amino acids content is in line with other reports suggesting complex effects of OsGS1-2 function (Bao et al., 2014). Consistent with our results, several Arabidopsis mutants affected in the regulation of nitrogen and amino acid metabolism have been shown to be impaired for pathogen resistance: *nrt2-1* (Camañes et al., 2012), *nrt2-6* (Dechorgnat et al., 2012), *amt1-1* (Pastor et al., 2014), *atl-31*, and *atl-6* (Maekawa et al., 2012). The Arabidopsis *lht1* mutant (lys histidine transporter 1) shows strong resistance to several pathogens that could be explained by a reduced cellular content in proline, glutamine and alanine (Liu et al., 2010). This is consistent with our observation that glutamine and alanine levels are increased in the case of enhanced susceptiblity in Kasalath plants under high fertilization (Supplementary Figure 2). Additional measurements are needed to establish whether modifications of metabolite fluxes and/or quantities are responsible for NIS. Infection by *M. oryzae* is known to modify the plant TCA cycle, in particular GABA-shunt, probably to divert the host metabolism (Wu et al., 2006; Takahashi et al., 2008). Based on our RNA-seq results (Figure 4), some key components of the TCA cycle that are differentially regulated (OsGAD3 and OsGDH3) could also participate to the fueling of the pathogen during infection under high nitrogen. Altogether, these elements support the hypothesis that nitrogen fertilization fuels growth of *M. oryzae*.

### Nitrogen fertilization impacts the pathogenicity program of *M. oryzae*

One final hypothesis could be that nitrogen fertilization could indirectly regulate fungal pathogenicity functions. Despite a large amount of data on *M. oryzae* effector and secretome (Matsumura et al., 2003; Mosquera et al., 2009; Kim et al., 2010; Mathioni et al., 2011; Kawahara et al., 2012), little is known on the regulation of *M. oryzae* effector by the physiological status of the plant. Recently, we have demonstrated that in rice plants recovering from drought, the expression of *M. oryzae* effectors was massively down-regulated (Bidzinski et al., 2016). More generally the regulation of plant pathogen effectors is still largely underscored (McCotter et al., 2016). We identified a set of *M. oryzae* genes regulated by the level of nitrogen fertilization applied to the host plant. Here we were able to demonstrate that *M. oryzae* effectors are more induced in nitrogen-fed Kasalath plants than in nitrogen-deprived ones, and thus that pathogenicity functions are potentially increased. However, according to available knowledge on fungus biology *in vitro*, a nitrogen-rich environment should turn down many pathogenicity genes (Donofrio et al., 2006; Wilson et al., 2012; Fernandez and Wilson, 2013). This apparently contradictory set of data could be explained by the fact that all previous studies were done *in vitro* whereas our present study was conducted *in planta*. Thus, NIS in Kasalath plants could be the consequence of an up-regulation of pathogen effectors under this condition. This is similar to the observation that in *Cladosporium fulvum*, the expression of the Avr9 effector is induced by nitrogen (Thomma et al., 2006). Our result thus extends this observation by showing that a massive re-programming of effectors can be controlled by nitrogen fertilization *in planta*.

To conclude, our results clearly indicate that nitrogen fertilization increases susceptibility despite an increase in the expression of several defense genes. This enhanced defense may be directly or indirectly due to an increase in some metabolites (e.g., Glutamine). At the meantime, the fungal pathogenicity program is also visibly up-regulated by nitrogen fertilization, although it is yet difficult to identify which metabolite is responsible for this behavior. We propose that after nitrogen fertilization, despite an increase in defense, the host does not succeed in facing a concomitant increase in pathogenicity of the fungus, leading to enhanced susceptibility The logic applied in this work allowed us to identify *GS1-2* gene as a potential key player in NIS. These results open the way to possible improvement of disease resistance under high fertilization.

## Author contributions

HH, TN, EB, and JM took care of the plants, inoculation, disease symptoms analysis and cytology. HH has performed RNA extractions and RT-QPCRs. HH and EB performed the statistical analyses. EB analyzed RNA-seq gene expression experiments with JM. SB and AG expertized the data for metabolome. JM and EB designed the experiments. EB drafted the manuscript. JM completed the draft. All authors read and approved the final manuscript.

## Funding

This work was supported by two PhD grants from the Vietnamese and Chinese governments. This work was supported by the GARP project from the ANR-SYSTERRA and MetaboHUB-ANR-11-INBS-0010 programs. Part of this work was supported by Natural Science Foundation of Yunnan Province of China (2016FD025).

### Conflict of interest statement

The authors declare that the research was conducted in the absence of any commercial or financial relationships that could be construed as a potential conflict of interest.

## Supplementary material

Supplementary Figure 1 **Plant growth under variable nitrogen regimes**. Plant height was measured before and during the infection of rice plants **(A)** (Nipponbare) and **(B)** (Kasalath) with the Guy11 isolate. Leaf elongation rate was calculated based on these growth measurements. Different nitrogen regimes (0N or 1N; see Section Materials and Methods) were applied 1 day before inoculation. The mean and standard deviation of three biological replicates are shown. A Student *t*-tests corrected by Bonferroni was used to compare the 0N and 1N conditions; ^*^*P* < 0.05.

Supplementary Figure 2 **Plant total nitrogen and metabolome content under variable nitrogen regimes**. Plant total nitrogen content was measured before and during the infection of rice plants **(A)** (Nipponbare) and **(B)** (Kasalath) with the Guy11 isolate. Metabolomics changes in leaves were measured before inoculation and 2 days after inoculation in Nipponbare **(C** and **E)** and Kasalath **(D** and **F)**. The measurements were done in three independent experiments. Metabolites concentrations were corrected by fresh weight and normalized between experiments. Only the 7 amino acids for which significant quantities were measured are shown. Different nitrogen regimes (0N or 1N; see Section Materials and Methods) were applied 1 day before inoculation. The mean and standard deviation of three biological replicates are shown. A Student *t*-test corrected by Bonferroni was used to compare the 0N and 1N conditions; ^*^*P* < 0.05.

Supplementary Figure 3 **Effect of nitrogen supply on lesion number, type and surface after rice blast infection of Nipponbare and Kasalath genotypes**. Different doses of nitrogen (0N, 1N; see Section Materials and Methods) were supplied to rice 1 day before inoculation with *M. oryzae* Guy11. **(A)** The number of susceptible lesions and necrotic like lesions were counted on six different leaves in three independent experiments. **(B)** The surface of individual susceptible lesions were measured (*n* = approx. 240 for each condition). ^**^Student test; *P* < 0.01; ^*^Student test; *P* < 0.05.

Supplementary Figure 4 **Nitrogen-induced susceptibility to several rice blast isolates in Kasalath and Nipponbare**. One day before inoculation, plants were treated with either 0N or 1N solutions (see Section Materials and Methods). Plants were inoculated with different *M. oryzae* isolates. Np, Nipponbare; Ka, Kasalath. The isolates used were CD203, CL32, Guy11, TH16, US31, and VT2. This experiment was repeated several times and gave similar results; one representative experiment is shown.

Supplementary Figure 5 **Expression of *OsGS1-2* in RNA-seq data**. Expression values were normalized and differential expression was tested with DESeq. Corrected expression values obtain after DESeq analysis are presented. *P*-value adjusted < 0,001.

Supplementary Figure 6 **Expression of *OsGS1-2* in T-DNA insertion line XFGSTJ. (A)** Schematic insertion of T-DNA in the mutant line XFGSTJ. **(B)** Expression of OsGS1-2 in different plants before inoculation. Homozygous plants could not be found, probably because this mutation is lethal, and only heterozygous (He) plants were analyzed. No difference in growth were observed on young plants in our experimental conditions, possibly because only heterozygous plants were obtained.

Supplementary Figure 7 **Metabolome of OsGS1-2 heterozygote T-DNA and wild-type lines**. Metabolomics changes in leaves were measured before inoculation in heterozygous *gs1-2* mutants and in the corresponding wild-type plants. The measures were done twice in one experiment. The log_2_ ratio between heterozygous *gs1-2* and wild-type is presented. A student *t*-test was used to compare mutant and wild type ^**^*P* < 0.01

Supplementary Figure 8 **Validation of RNA-seq for *M. oryzae* pathogenicity genes by quantitative RT-PCR**.

Supplementary Table 1 **Primers used in the qRT-PCR analysis**.

Supplementary Table 2 **List of 243 rice DEG gene at 0 dpi**.

Supplementary Table 3 **List of the 2,665 rice DEG at 2 dpi**.

Supplementary Table 4 **Enrichment analysis for rice genes significantly express at 2 dpi**. In each gene set, the frequency of gene with a selected known annotation has been calculated. The enrichment in these functions was tested by a chi2 analysis corrected by Bonferroni. ^***^*P* < 0.001, ^**^*P* < 0.01, ^*^*P* < 0.5 compared with the total database gene set. A, enrichment analysis with arrayexpress database with GO ontologies.

Supplementary Table 5 **List of 3428 *M. oryzae* genes significantly express at 2 dpi**.

Supplementary Table 6 **Enrichment analysis for *M. oryzae* gene expressed in planta at 2 dpi**. ^**^*P* < 0.01; ^***^*P* < 0.001 Chi2 analysis corrected with Bonferroni

Supplementary Table 7 **24 *M. oryzae* DEG gene at 2 dpi between 0N and 1N condition**. Differential expression between NIS and no NIS repetition was performed on DEB website (Yao and Yu, 2011). For Differentially expressed gene analysis we used two different statistical analysis DESeq and EdgeR with a minimum FDR of 5%. We considered as differentially expressed genes all genes that are significant in at least one of the statistical test.

## Abbreviations

0N: Fertilization without nitrogen
1N: Fertilization with nitrogen
DEG: Differentially expressed gene
dpi: day post inoculation
NIS: Nitrogen-Induced Susceptibility.
