# Supplementary figures and images for: Increase of Fungal Pathogenicity and Role of Plant Glutamine in Nitrogen-Induced Susceptibility (NIS) To Rice Blast
Source: Front Plant Sci. 2017 Feb 28;8:265. doi: 10.3389/fpls.2017.00265 (PMC5329020; doi:10.3389/fpls.2017.00265)

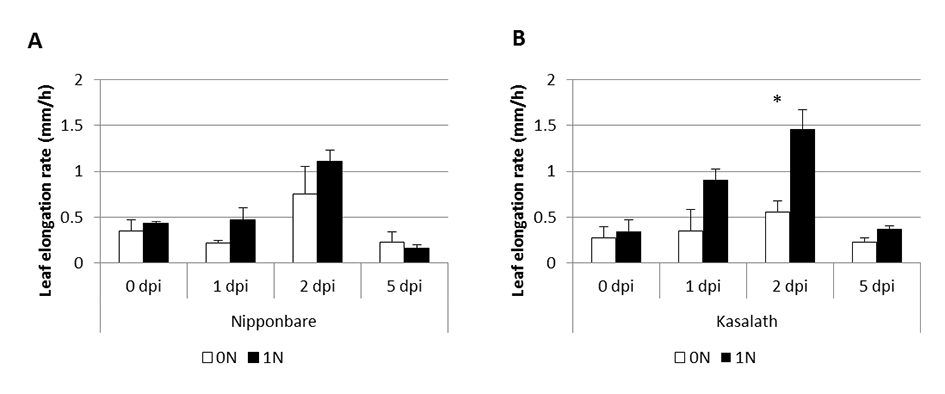

Supplement: Supplementary Figure 1 — Plant growth under variable nitrogen regimes. Plant height was measured before and during the infection of rice plants (A) (Nipponbare) and (B) (Kasalath) with the Guy11 isolate. Leaf elongation rate was calculated based on these growth measurements. Different nitrogen regimes (0N or 1N; see Section Materials and Methods) were applied 1 day before inoculation. The mean and standard deviation of three biological replicates are shown. A Student t-tests corrected by Bonferroni was used to compare the 0N and 1N conditions; *P < 0.05. [file Image1.TIF]

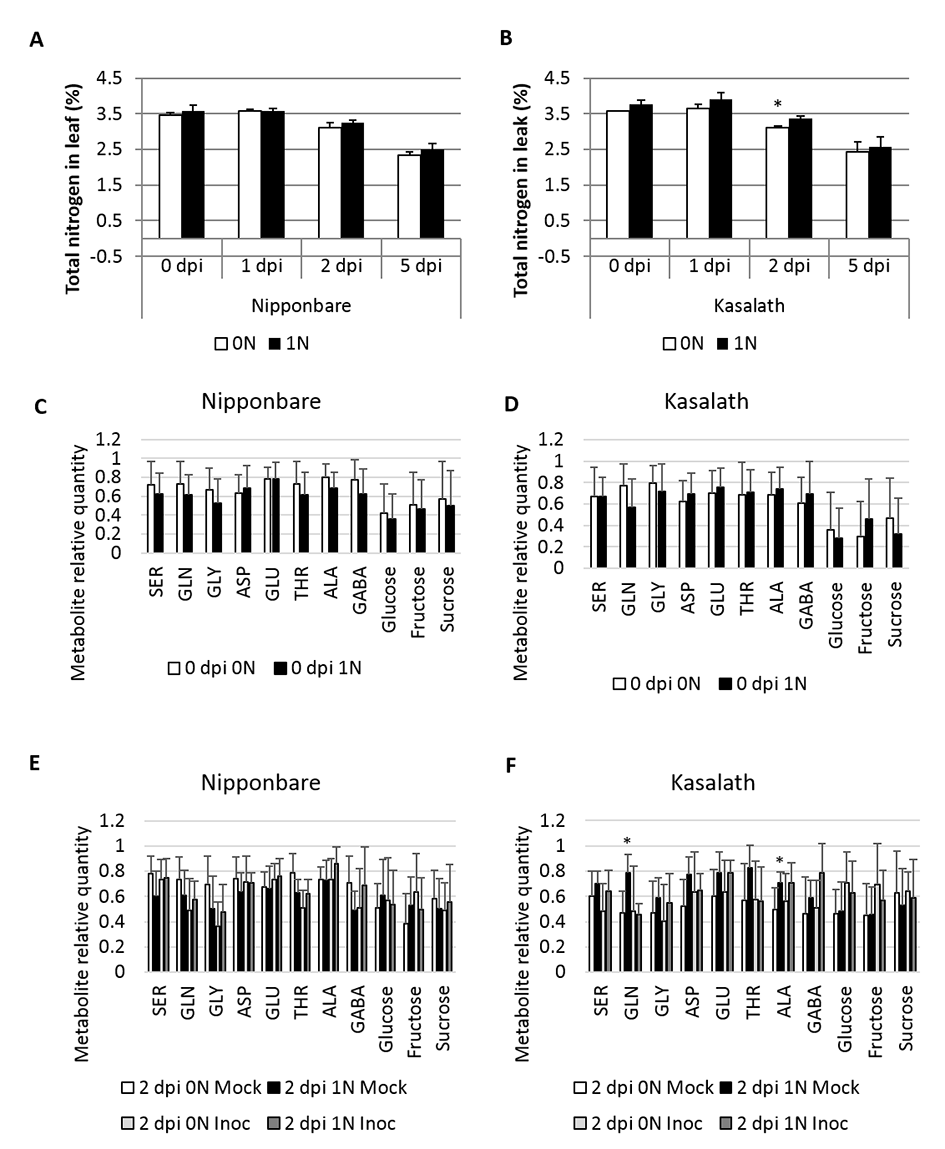

Supplement: Supplementary Figure 2 — Plant total nitrogen and metabolome content under variable nitrogen regimes. Plant total nitrogen content was measured before and during the infection of rice plants (A) (Nipponbare) and (B) (Kasalath) with the Guy11 isolate. Metabolomics changes in leaves were measured before inoculation and 2 days after inoculation in Nipponbare (C and E) and Kasalath (D and F). The measurements were done in three independent experiments. Metabolites concentrations were corrected by fresh weight and normalized between experiments. Only the 7 amino acids for which significant quantities were measured are shown. Different nitrogen regimes (0N or 1N; see Section Materials and Methods) were applied 1 day before inoculation. The mean and standard deviation of three biological replicates are shown. A Student t-test corrected by Bonferroni was used to compare the 0N and 1N conditions; *P < 0.05. [file Image2.TIF]

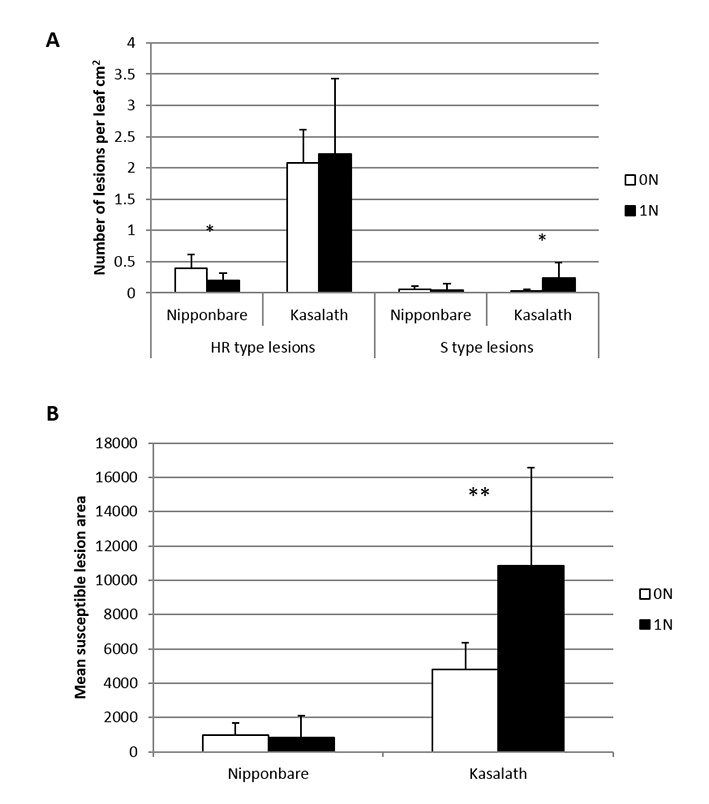

Supplement: Supplementary Figure 3 — Effect of nitrogen supply on lesion number, type and surface after rice blast infection of Nipponbare and Kasalath genotypes. Different doses of nitrogen (0N, 1N; see Section Materials and Methods) were supplied to rice 1 day before inoculation with M. oryzae Guy11. (A) The number of susceptible lesions and necrotic like lesions were counted on six different leaves in three independent experiments. (B) The surface of individual susceptible lesions were measured (n = approx. 240 for each condition). **Student test; P < 0.01; *Student test; P < 0.05. [file Image3.TIF]

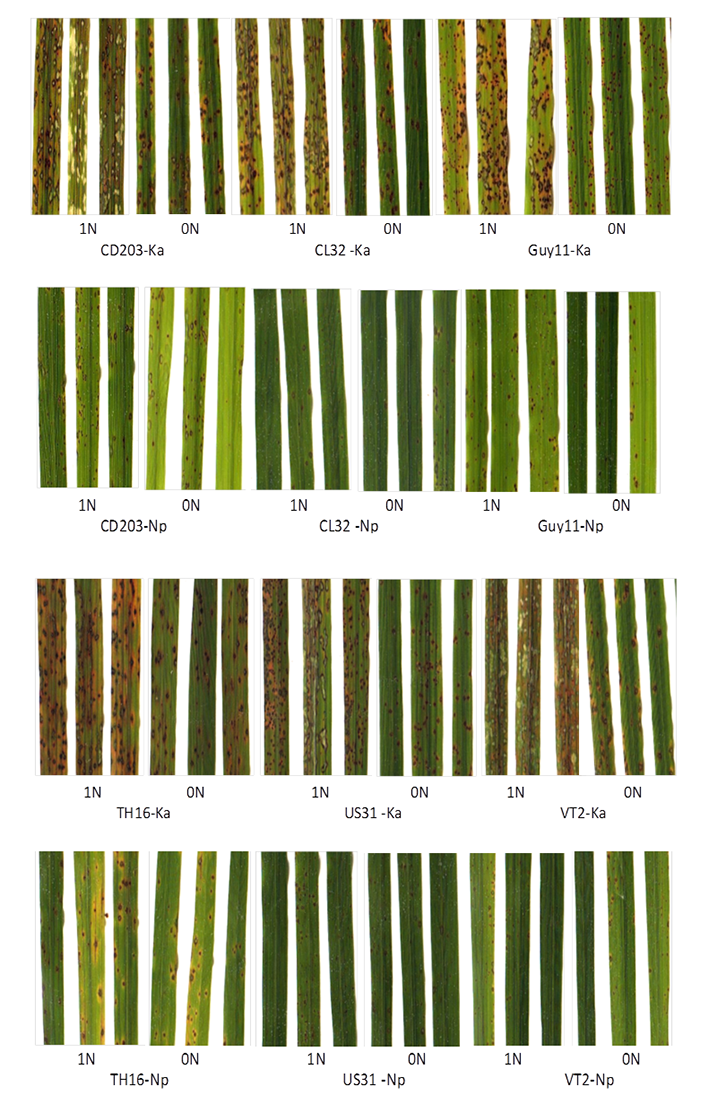

Supplement: Supplementary Figure 4 — Nitrogen-induced susceptibility to several rice blast isolates in Kasalath and Nipponbare. One day before inoculation, plants were treated with either 0N or 1N solutions (see Section Materials and Methods). Plants were inoculated with different M. oryzae isolates. Np, Nipponbare; Ka, Kasalath. The isolates used were CD203, CL32, Guy11, TH16, US31, and VT2. This experiment was repeated several times and gave similar results; one representative experiment is shown. [file Image4.TIF]

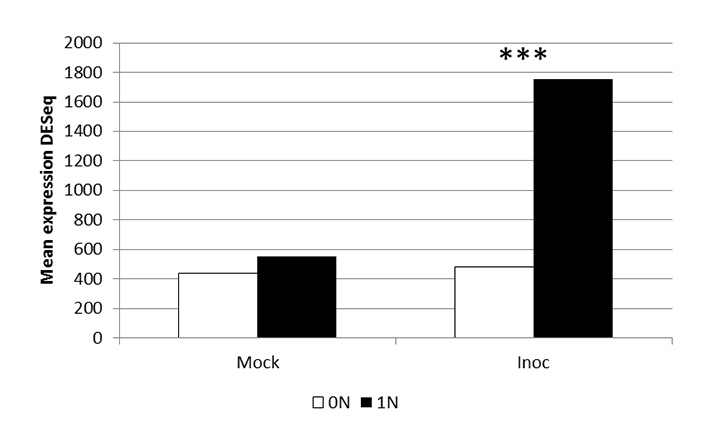

Supplement: Supplementary Figure 5 — Expression of OsGS1-2 in RNA-seq data. Expression values were normalized and differential expression was tested with DESeq. Corrected expression values obtain after DESeq analysis are presented. P-value adjusted < 0,001. [file Image5.TIF]

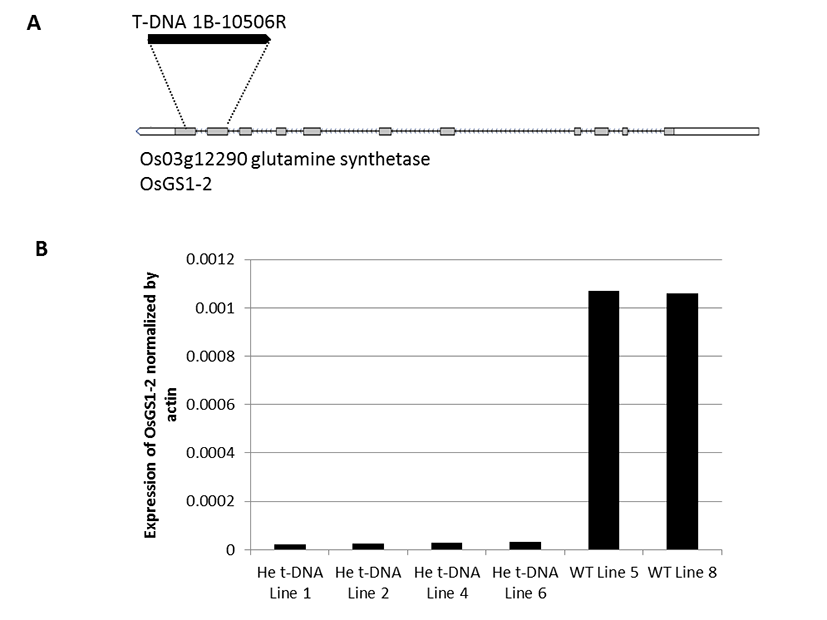

Supplement: Supplementary Figure 6 — Expression of OsGS1-2 in T-DNA insertion line XFGSTJ. (A) Schematic insertion of T-DNA in the mutant line XFGSTJ. (B) Expression of OsGS1-2 in different plants before inoculation. Homozygous plants could not be found, probably because this mutation is lethal, and only heterozygous (He) plants were analyzed. No difference in growth were observed on young plants in our experimental conditions, possibly because only heterozygous plants were obtained. [file Image6.TIF]

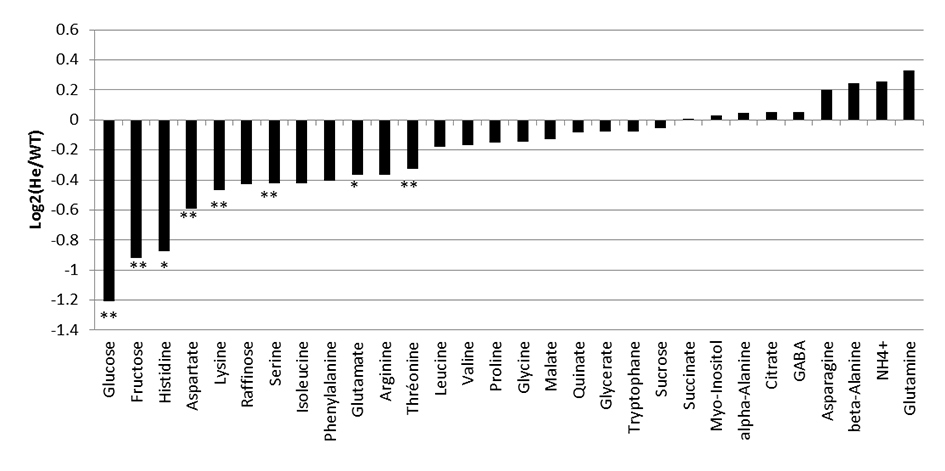

Supplement: Supplementary Figure 7 — Metabolome of OsGS1-2 heterozygote T-DNA and wild-type lines. Metabolomics changes in leaves were measured before inoculation in heterozygous gs1-2 mutants and in the corresponding wild-type plants. The measures were done twice in one experiment. The log2 ratio between heterozygous gs1-2 and wild-type is presented. A student t-test was used to compare mutant and wild type **P < 0.01 [file Image7.TIF]

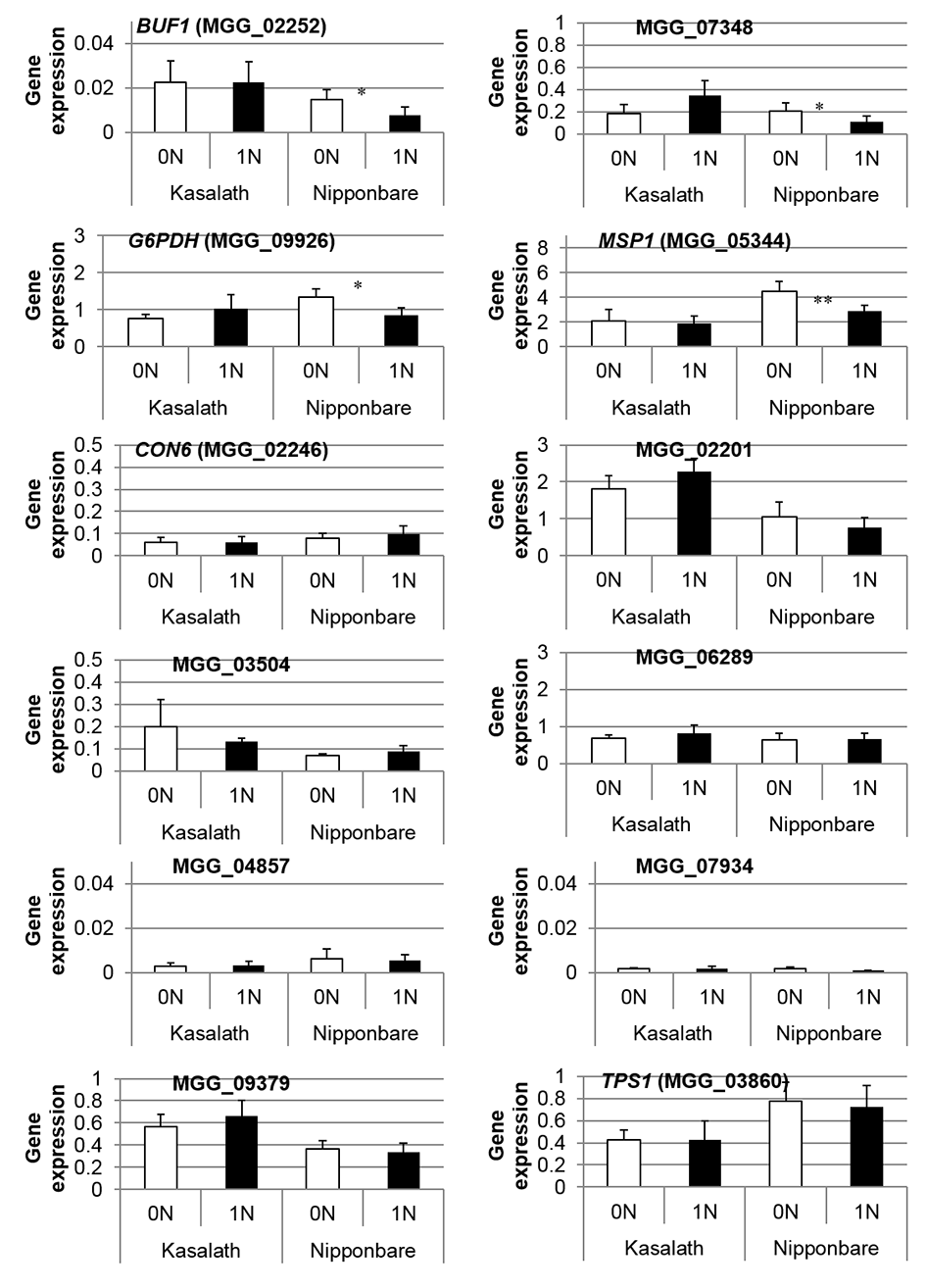

Supplement: Supplementary Figure 8 — Validation of RNA-seq for M. oryzae pathogenicity genes by quantitative RT-PCR. [file Image8.TIF]
